# Causal Effects of Genetically Determined Metabolites on Risk of Polycystic Ovary Syndrome: A Mendelian Randomization Study

**DOI:** 10.3389/fendo.2020.00621

**Published:** 2020-09-08

**Authors:** Shuliu Sun, Minjie Jiao, Chengcheng Han, Qian Zhang, Wenhao Shi, Juanzi Shi, Xiaojuan Li

**Affiliations:** ^1^Department of Obstetrics and Gynecology, Northwest Women's and Children's Hospital, Xi'an, China; ^2^The Assisted Reproductive Centre, Northwest Women's and Children's Hospital, Xi'an, China

**Keywords:** genetically determined metabolites, polycystic ovary syndrome, mendelian randomization, epiandrosterone sulfate, rs13222543

## Abstract

**Background:** Polycystic ovary syndrome (PCOS) is a heterogeneous endocrine disorder that is influenced by both genetic and environmental factors. However, the etiology of PCOS remains unclear.

**Methods:** We conducted a two-sample Mendelian randomization (MR) analysis to assess the causal effects of genetically determined metabolites (GDMs) on the risk of PCOS. We used summary level data of a genome-wide association study (GWAS) on 486 metabolites (*n* = 7,824) as exposure and a PCOS GWAS consisting of 4,138 cases and 20,129 controls as the outcome. Both datasets were obtained from publicly published databases. For each metabolite, a genetic instrumental variable was generated to assess the relationship between the metabolite and PCOS. For MR analysis, we primarily used the standard inverse variance weighted (IVW) method, while three additional methods—the MR-Egger, weighted median, and MR-PRESSO (pleiotropy residual sum and outlier) methods—were performed as sensitivity analyses.

**Results:** Using genetic variants as predictors, we observed a robust relationship between epiandrosterone sulfate (EPIA-S) and PCOS (*P*_IVW_ = 0.0186, *P*_MR−Egger_ = 0.0111; *P*_Weighted−median_ = 0.0154, and *P*_MR−PRESSO_ = 0.0290). Similarly, 3-dehydrocarnitine, 4-hydroxyhippurate, hexadecanedioate, and β-hydroxyisovalerate may also have causal effects on PCOS development.

**Conclusions:** We identified metabolites that might have causal effects on PCOS development. Our study emphasizes the role of genetic factors underlying the causal relationships between metabolites and PCOS and provides novel insights through the integration of metabolomics and genomics to better understand the mechanisms involved in human disease pathogenesis.

## Introduction

Polycystic ovary syndrome (PCOS) is the leading cause of female infertility worldwide, affecting 6–20% of the female population of reproductive age (1, 2). Hyperandrogenism (HA), ovulatory dysfunction (OD), and polycystic ovarian morphology (PCOM) are common clinical manifestations of PCOS (3). Furthermore, women with PCOS usually exhibit a wide range of endocrine-metabolic disturbances, including insulin resistance, hyperinsulinemia, obesity, and adipose tissue dysfunction, which eventually result in type 2 diabetes mellitus and cardiovascular disease (4–6). Although the latest advances have suggested that PCOS is a complex disease influenced by both genetic and environmental factors, its etiology and the underlying biological processes still need to be researched.

Modern omics-based technologies, including genomics, transcriptomics, proteomics, and metabolomics, have greatly advanced our understanding of the pathophysiological process of human complex diseases and successfully identified a series of biomarkers that could lead to earlier diagnosis of diseases or therapeutic targets for disorders (7). Therefore, these approaches provide a systematic readout of the inherent genetic architecture, the dynamics of physiological and biochemical indicators, and the environmental exposure for individuals (8). Metabolomics characterize downstream gene regulation and protein activity and are considered to be more representative of clinical phenotypes. During the past decade, advances in metabolomics have led to considerable achievements in detecting chemical components that contribute to the occurrence of PCOS. Zhao et al. assessed the metabolic profiles of 217 cases and 48 controls and identified a series of carbohydrate, lipid, and amino acid metabolism in PCOS (9). Chang et al. performed a combinative analysis of non-targeted and targeted metabolomics on obese women and found specific amino acid elevations in PCOS (10). Zhang et al. recruited 286 subjects and investigated the disturbed metabolic profiles for specific pathogenic characteristics of PCOS patients, such as HA and insulin resistance (11). However, these studies typically had small sample sizes and provided limited information about pathophysiological mechanisms. A comprehensive analysis of genomics and metabolomics could provide novel insights into understanding the underlying mechanism of genetic and metabolic interactions in the pathogenesis of PCOS. Recently, a database of genotype-dependent metabolic phenotypes [called genetically determined metabolites (GDMs)] has been developed using a genome-wide association study (GWAS) with non-targeted metabolomics. The established GDMs provide functional intermediates to facilitate understanding of the potential relevance of human serum metabolites and related genetic variants in the pathogenesis of complex diseases (12–15).

Mendelian randomization (MR) is a novel genetic epidemiological approach that uses genetic variants as instrumental variables to assess the causality of an agent on clinical outcomes of interest (16). The basic principle of the MR study design uses instrumental variables rather than only exposure to infer causality of exposures on clinical outcomes. This primarily requires the assumption that the generated instrumental variable (usually genetic variants) is reliably associated with the exposure and acts on the outcome directly through exposure of interest. Unlike traditional metabolomic approaches, MR can provide unbiased detection of causal effects, considering the fact that genetic variants are less susceptible to environmental factors (17, 18). In the past decade, MR has been widely applied to infer causal relationships using publicly available GWAS summary statistics (19–21). Taking advantage of GDMs and GWAS findings for PCOS, we conducted this two-sample (exposure and outcome measured in different samples) MR study to (i) assess the causal effects of 486 serum metabolites on the risk of developing PCOS and (ii) investigate the genetic variants that determine the variation of the metabolites, which also contribute to the development of PCOS.

## Materials and Methods

### Genome-Wide Association Study of Serum Metabolites

We obtained data for genetic factors that influence human blood metabolites from the study of Shin et al. (15). They conducted genome-wide association scans using the metabolome as a phenotype. The study comprised 7,824 adult individuals from two European population studies. Metabolic profiling was performed on fasting serum using ultrahigh-performance liquid-phase chromatography and gas chromatography coupled with tandem mass spectrometry (13, 22). A standardized process of identification and relative quantification, data reduction, and quality assurance was performed using Metabolon, Inc. (https://www.metabolon.com/). A total of 486 metabolites, assigned to eight broad metabolic groups (amino acids, carbohydrates, cofactors and vitamins, energy, lipids, nucleotides, peptides, and xenobiotic metabolism), were included in the final GWAS analysis. Among these, 196 (37%) were classified as “unknown,” which meant that their chemical identity had not been clearly determined thus far. We included these “unknown” metabolites in our study as they still attracted attention from other researchers and might provide further useful information in the future (22). After genotyping, imputation, and quality control (QC), ~2.1 million single-nucleotide polymorphisms (SNPs) were identified in the final GWAS meta-analysis. The complete GWAS summary statistics are publicly available through the Metabolomics GWAS server at http://metabolomics.helmholtz-muenchen.de/gwas/.

### Genetic Instrumental Variables for 486 Metabolites

In order to satisfy the primary conditions for preforming MR, we implemented strict procedures to select the genetic instruments of these 486 metabolites. First, we screened out the genetic variants that were strongly (*P* < 1 × 10^−5^) associated with specific metabolites to ensure that the generated instrument could explain a larger variance in the corresponding metabolite. Next, we selected independent SNPs (r^2^ < 0.1 within ± 500 kb) to generate the instrumental variable using a clumping procedure with the European 1,000G as reference panel. We further tested whether these genetic instruments could explain the variation of the corresponding metabolites to avoid instruments with a weak first stage. The proportion of variability (*R*^2^) and F statistic was calculated to assess the strength of these instrumental variables (23). F statistic > 10 was considered for selection of strong instrumental variables (24).

### Genome-Wide Association Study of Polycystic Ovary Syndrome

Genetic associations with PCOS were obtained from a recent large GWAS meta-analysis, with 4,138 cases and 20,129 controls collated from six European cohorts (25). The included samples were either diagnosed according to the National Institutes of Health (NIH) (require HA and OD) or Rotterdam criteria (requires at least two traits of HA, OD, and PCOM) (26). All data involved in the GWAS analysis had been approved by the authors' Institutional Review Board (IRB). Written informed consent was also obtained from all participants. Summary-level results were obtained from these studies, and QC procedures were performed according to the EasyQC pipeline (27). The genome-wide association analysis was performed using a fixed-effect, inverse variance weighted (IVW) meta-analysis using METAL (28).

### Statistical Analysis

To calculate causal estimates, we used the standard IVW method for the two-sample MR analysis of the summarized datasets of the serum metabolites and PCOS (16). The IVW approach was employed with the fundamental assumption that all genetic variants referred to valid instruments, and it thus provided efficient and consistent casual estimates. Specifically, the IVW estimate could be equivalently interpreted as a liner regression with SNP–exposure associations as the independent variable and SNP–outcome associations as the dependent variable, setting the intercept term to zero. The *P*-value was calculated from a standard normal cumulative distribution function of the ratio of the combined causal effects and its standard error. The results were considered statistically significant at the threshold of *P* < 0.05.

The IVW approach referred to the primary MR analysis and successfully aided in inferring the causality of an exposure for outcome. However, there were still several concerns. One important concern was the existence of horizontal pleiotropy. Horizontal pleiotropy occurs when any variants were invalid instruments and acted on the outcome through other ways (not through the concerned exposure). To control for horizontal pleiotropy, we next applied additional MR methods for sensitivity analyses: the weighted median method, which allowed a subset of genetic variants (<50%) to be invalid instrumental variables (29); MR-Egger, which worked even when up to 50% of the variants came from invalid instrumental variables (30); and MR-PRESSO (pleiotropy residual sum and outlier), which could provide a pleiotropy residual sum and outlier test by identifying and discarding horizontal pleiotropic outliers (31). Further, we detected the presence of horizontal pleiotropy through the MR-PRESSO Global test. All MR analyses were carried out using the R package “MendelianRandomisation” as well as the MR-PRESSO software (https://github.com/rondolab/MR-PRESSO).

## Results

### Genetic Instruments for 486 Metabolites

Following standard procedures, we obtained the instrumental variables containing 3–675 independent SNPs for the 486 metabolites. The variables explained 0.8–83.5% (median 4.7%) of the variance for their corresponding metabolites. The minimum F statistic for representing the strength of the predictive instrumental variable was 20.33. All instrumental variables for the 486 metabolites were sufficiently informative for MR analysis.

### Causal Effects of 486 Metabolites on Polycystic Ovary Syndrome

**Figure 1:**
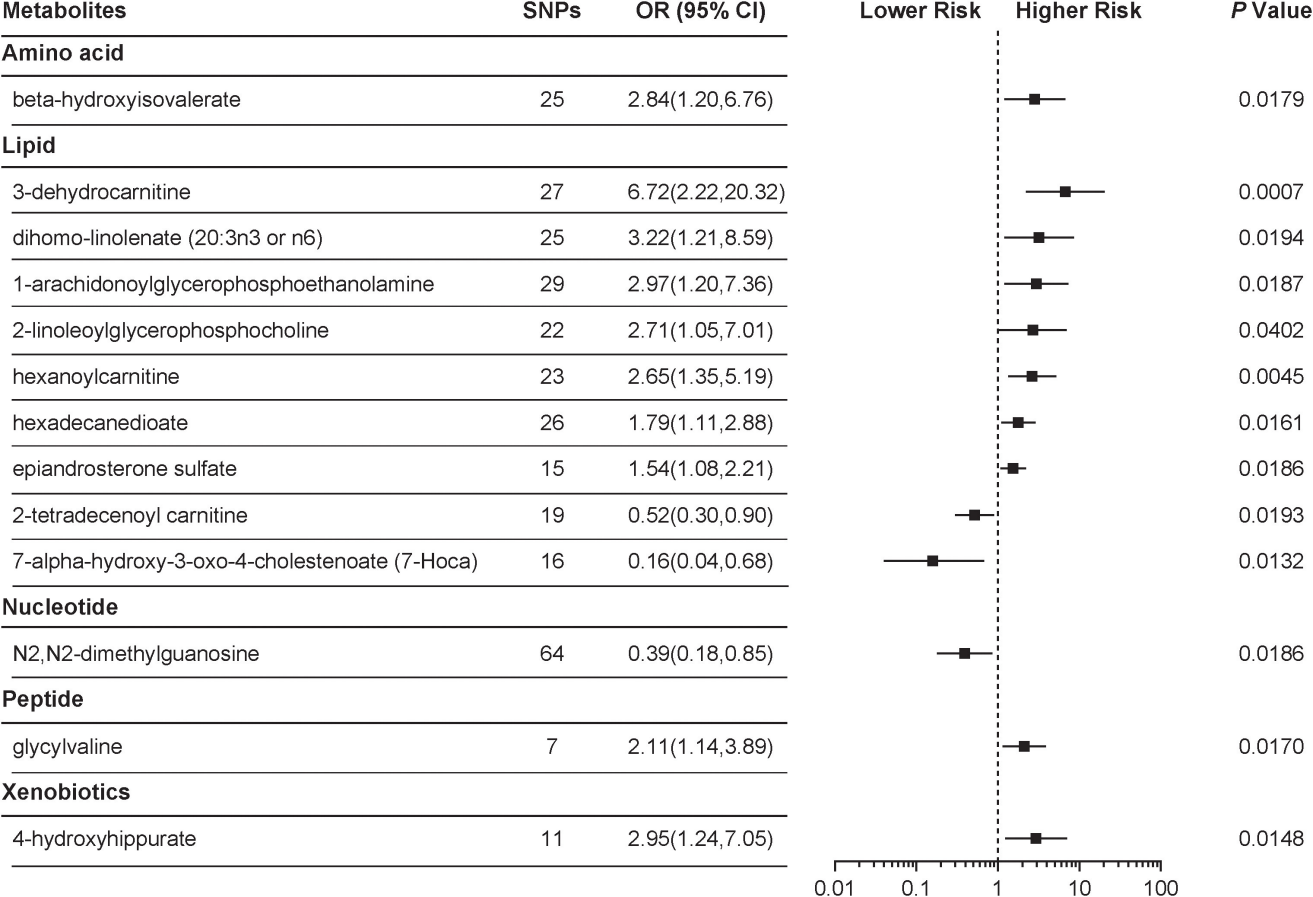
Mendelian randomization (MR) association of serum metabolites on the risk of polycystic ovary syndrome (PCOS). Causal estimates are obtained using metabolite-related single-nucleotide polymorphisms (SNPs) as instrumental variables using the inverse variance weighted (IVW) method. Odds ratio (OR) and 95% confidence intervals (95% CIs) are provided for PCOS per 1-s.d. higher level of specific metabolite.

With the use of genetic variants as proxies, the IVW identified 24 metabolites with causal effects on PCOS, among which 13 (54.2%) were known metabolites while the remaining 11 belonged to the “unknown” subgroup (Figure 1, Supplemental Table 1). We focused on the 13 known metabolites, which included nine lipids, a xenobiotic, a peptide, an amino acid, and a nucleotide. 3-Dehydrocarnitine was the most significant chemical compound with predicted causal effects on PCOS (*P* = 0.0007). The risk of developing PCOS increased 5-fold for a 1-s.d. increase in the level of 3-dehydrocarnitine (OR = 6.72; 95% CI 2.22–20.32). Two other carnitines with causal associations with PCOS were hexanoylcarnitine (OR = 2.65; 95% CI 1.35–5.19; *P* = 0.0045) and 2-tetradecenoyl carnitine (OR = 0.52; 95% CI 0.30–0.90; *P* = 0.0193). Notably, 2-tetradecenoyl carnitine had an inverse association with PCOS, unlike 3-dehydrocarnitine and hexanoylcarnitine. 4-Hydroxyhippurate, a xenobiotic, was associated with an increased risk of developing PCOS. β-Hydroxyisovalerate, classified as an amino acid, also appeared to be a pathogenic risk factor for PCOS (OR = 2.84; 95% CI 1.20–6.76; *P* = 0.0179).

### Sensitivity Analysis

**Figure 2:**
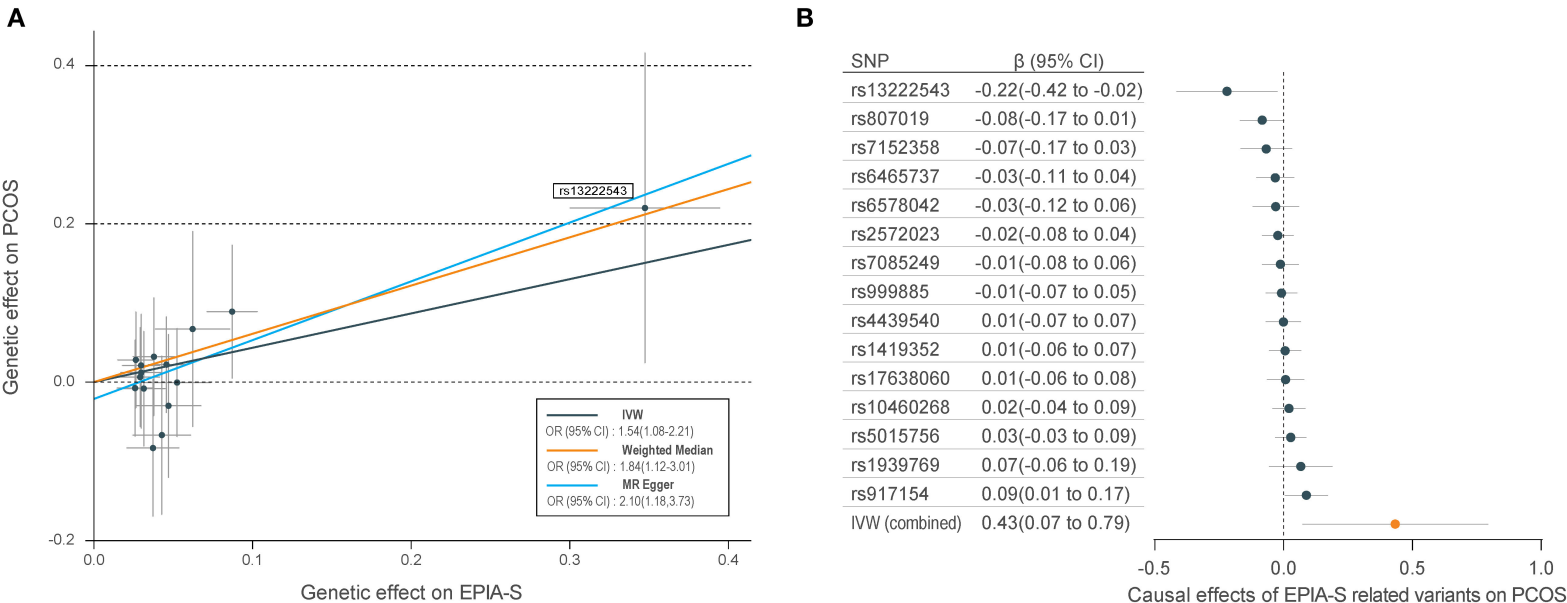
Mendelian randomization (MR) plots for relationship of epiandrosterone sulfate (EPIA-S) with polycystic ovary syndrome (PCOS). **(A)** Scatter plot of potential effects of single-nucleotide polymorphisms (SNPs) on EPIA-S vs. PCOS, with the slope of each line corresponding to the estimated MR effect per method. SNPs showing negative signals with EPIA-S are plotted after orientation to the exposure-increasing allele. SNPs with higher effects on both metabolites and PCOS are marked on the plots. **(B)** Forest plot of individual and combined effects of EPIA-S related SNPs on PCOS. Data are expressed as raw β values with 95% confidence interval (CI).

**Figure 3:**
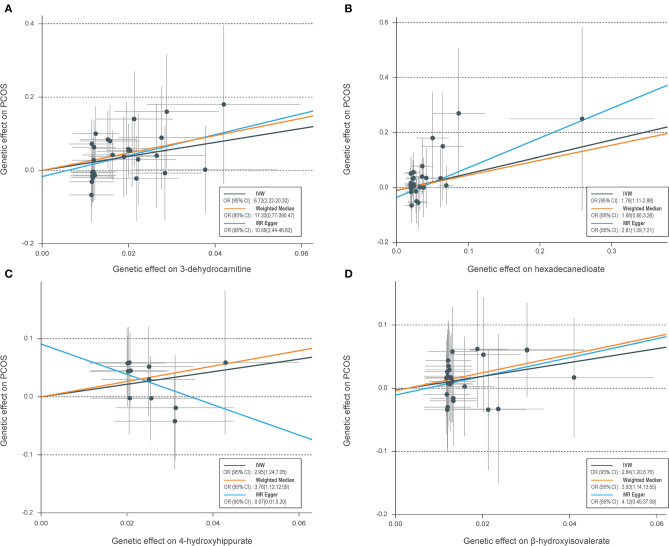
Scatter plots of genetic associations with four suggestive metabolites vs. the associations with polycystic ovary syndrome (PCOS). **(A)** 3-Dehydrocarnitine; **(B)** hexadecanedioate; **(C)** 4-hydroxyhippurate; **(D)** β-hydroxyisovalerate. Each of the single-nucleotide polymorphisms (SNPs) associated with metabolites are represented by a black dot with the error bar depicting the standard error of its association with metabolite (horizontal) and PCOS (vertical). The slopes of each line represent the causal association for each method. SNPs showing negative signals with metabolites are plotted after orientation to the exposure-increasing allele.

Table 1 shows the sensitivity analysis results for assessing the robustness of our IVW estimates. Epiandrosterone sulfate (EPIA-S) was the only metabolite with robust associations across all additional MR methods (*P*_IVW_ = 0.0186; *P*_MR−Egger_ = 0.0111; *P*_Weighted−median_ = 0.0154; and *P*_MR−PRESSO_ = 0.0290), and there was no evidence of horizontal pleiotropy (*P*_Global_ = 0.5190). Using 15 genetic predictors as instrumental variables (variance explained = 8.0%; F statistic = 45.53), we observed a 50% higher risk of developing PCOS for each 1-s.d. increase in the level of EPIA-S (Figure 2A). There were also several metabolites that passed some of the additional sensitivity tests, such as 3-dehydrocarnitine (*P*_MR−Egger_ = 0.0727; *P*_Weighted−median_ = 0.0017; and *P*_MR−PRESSO_ = 0.0023, Figure 3A), 4-hydroxyhippurate (*P*_MR−Egger_ = 0.2294; *P*_Weighted−median_ = 0.0318; and *P*_MR−PRESSO_ = 0.0351, Figure 3B), hexadecanedioate (*P*_MR−Egger_ = 0.0320; *P*_Weighted−median_ = 0.1272; and *P*_MR−PRESSO_ = 0.0238, Figure 3C), and β-hydroxyisovalerate (*P*_MR−Egger_ = 0.2096; *P*_Weighted−median_ = 0.0300; and *P*_MR−PRESSO_ = 0.0051, Figure 3D). These metabolites may have plausible effects on PCOS because the MR-Egger and weighted median method are based on assumptions that might be incorrect. The relationship between hexadecanedioate and PCOS should be carefully investigated as the MR-Egger method yielded an inverse association compared to the other MR methods.

**Table 1:** Sensitivity analysis of causal associations between metabolites and PCOS.

**Metabolites**	**MR-Egger**		**Weighted median**		**MR-PRESSO**		**MR-PRESSO globle test**	
	**OR (95% CI)**	***P*-value**	**OR (95% CI)**	***P*-value**	**OR (95% CI)**	***P*-value**	**RSS**	***P*-value**
**Amino acid**								
Beta-hydroxyisovalerate	4.12 (0.45, 37.68)	0.2096	3.93 (1.14, 13.55)	**0.0300**	2.84 (1.46, 5.53)	**0.0051**	15.37	0.9477
**Lipid**								
3-Dehydrocarnitine	17.33 (0.77, 390.47)	0.0727	10.69 (2.44, 46.82)	**0.0017**	6.72 (2.22, 20.32)	**0.0023**	34.58	0.2115
Dihomo-linolenate (20:3n3 or n6)	19.51 (0.85, 449.74)	0.0635	3.16 (0.73, 13.57)	0.1223	3.22 (1.25, 8.32)	**0.0236**	24.31	0.5654
1-Arachidonoylglycerophosphoethanolamine	2.12 (0.05, 94.91)	0.6976	2.02 (0.54, 7.63)	0.2974	2.97 (1.20, 7.36)	**0.0259**	30.38	0.4551
2-Linoleoylglycerophosphocholine	31.89 (0.97, 1045.31)	0.0518	1.93 (0.50, 7.40)	0.3391	2.71 (1.18, 6.20)	**0.0283**	17.47	0.7762
Hexanoylcarnitine	3.50 (0.76, 16.1)	0.1076	2.35 (0.92, 6.00)	0.0735	2.65 (1.35, 5.19)	**0.0094**	24.80	0.4460
Hexadecanedioate	2.81 (1.09, 7.21)	**0.0320**	1.68 (0.86, 3.28)	0.1272	1.79 (1.11, 2.88)	**0.0238**	28.95	0.3879
Epiandrosterone sulfate	2.10 (1.18, 3.73)	**0.0111**	1.84 (1.12, 3.01)	**0.0154**	1.54 (1.09, 2.19)	**0.0290**	15.17	0.5190
2-Tetradecenoyl carnitine	0.46 (0.16, 1.33)	0.1498	0.53 (0.25, 1.11)	0.0935	0.52 (0.34, 0.82)	**0.0107**	13.09	0.8723
7-Alpha-hydroxy-3-oxo-4-cholestenoate (7-Hoca)	0.03 (0.01, 3.10)	0.1358	0.21 (0.03, 1.56)	0.1284	0.16 (0.05, 0.58)	**0.0133**	13.38	0.7102
**Nucleotide**								
N2,N2-Dimethylguanosine	0.20 (0.04, 1.11)	0.0653	0.40 (0.11, 1.50)	0.1751	0.39 (0.18, 0.84)	**0.0185**	61.28	0.6210
**Peptide**								
Glycylvaline	1.66 (0.11, 26.06)	0.7167	1.98 (0.86, 4.54)	0.1080	2.11 (1.20, 3.70)	**0.0407**	6.86	0.5691
**Xenobiotics**								
4-Hydroxyhippurate	0.07 (0.01, 5.20)	0.2294	3.76 (1.12, 12.59)	0.0318	2.95 (1.24, 7.05)	**0.0351**	12.29	0.4479

*Significant results for MR estimates (P < 0.05) are shown in bold. PCOS, polycystic ovary syndrome; MR, Mendelian randomization; PRESSO, pleiotropy residual sum and outlier*.

### Genetic Variants for Determining the Relationship Between Metabolites and Polycystic Ovary Syndrome

We further reported the potential genetic variants that might have decisive roles in determining the causal relationships between the metabolites and PCOS. Among the 15 SNPs in the instrumental variable of EPIA-S, rs13222543 showed the most significant association signal and the largest association coefficient with EPIA-S (β = −0.347; SE = 0.024; *P* = 3.31E−47, Table 2). Interestingly, it also showed a strong effect on PCOS (β = −0.220; SE = 0.100; *P* = 0.033, Figure 2B). Table 2 shows all the leading SNPs for determining the relationships for metabolites with PCOS. Further, we listed all the genetic variants for determining levels of EPAS-S, 3-dehydrocarnitine, 4-hydroxyhippurate, hexadecanedioate, β-hydroxyisovalerate, and the other metabolites in Supplemental Tables 2–7. These generated SNPs could provide important information for revealing potential pathophysiological mechanism or therapeutic targets for PCOS.

**Table 2:** Leading SNPs of the identified metabolites and their associations with PCOS.

**Metabolites**	**Leading SNP**	**Gene**	**For metabolite**			**For PCOS**		
			**β**	**SE**	***P*-value**	**β**	**SE**	***P*-value**
**Amino acid**								
Beta-hydroxyisovalerate	rs7720978	–	0.030	0.007	7.51E−06	0.061	0.038	0.110
**Lipid**								
3-Dehydrocarnitine	rs6691848	ZMYM6	0.029	0.006	2.12E−06	0.160	0.079	0.042
Dihomo-linolenate (20:3n3 or n6)	rs4978407	PALM2	0.023	0.005	5.90E−06	0.180	0.062	0.004
1-Arachidonoylglycerophosphoethanolamine	rs39741	CACNA2D1	−0.020	0.004	4.51E−06	−0.110	0.056	0.047
2-Linoleoylglycerophosphocholine	rs17160851	PDE1C	0.034	0.007	2.051E−06	0.120	0.083	0.160
Hexanoylcarnitine	rs17304141	SLC44A5	−0.128	0.012	2.46E−26	−0.1100	0.120	0.360
Hexadecanedioate	rs7926241	LOC105376595	0.087	0.019	3.56E−06	0.2700	0.120	0.024
Epiandrosterone sulfate	rs13222543	ZCWPW1	−0.347	0.024	3.31E−47	−0.220	0.100	0.033
2-Tetradecenoyl carnitine	rs6899136	DOCK2	0.035	0.008	7.35E−06	−0.1200	0.053	0.026
7-Alpha-hydroxy-3-oxo-4-cholestenoate (7-Hoca)	rs6755317	VWA3B	0.036	0.008	2.03E−06	−0.2100	0.110	0.042
**Nucleotide**								
N2,N2-Dimethylguanosine	rs16986182	DLGAP4	−0.128	0.027	1.81E−06	0.250	0.160	0.120
**Peptide**								
Glycylvaline	rs17093208	OTX2-AS1	−0.067	0.014	3.11E−06	−0.0780	0.048	0.100
**Xenobiotics**								
4-Hydroxyhippurate	rs4587865	LOC105370513	0.043	0.009	1.27E−06	0.0590	0.063	0.350

*SNP, single-nucleotide polymorphism; polycystic ovary syndrome*.

## Discussion

We performed a two-sample MR analysis to provide an unbiased detection of potential causal effects of GDMs on PCOS. Using genetic variants as proxies, we observed that genetically determined higher level of EPIA-S is associated with increased risk of developing PCOS. Our study also detected other metabolites that showed significant signals in most MR methods, including 3-dehydrocarnitine, 4-hydroxyhippurate, hexadecanedioate, and β-hydroxyisovalerate. Our findings screened out the potential genetic variants that contribute to the underlying causality of metabolites on PCOS. To the best of our knowledge, this is the first study integrating metabolomics with genomics to reveal the pathophysiological mechanisms of PCOS. Our study provides novel insights into the understanding of the role of interactions between genetic and metabolic factors in the pathogenesis of human diseases.

Based on 15 genetic scores with different degrees of specificity to EPIA-S, we demonstrated that high levels of EPIA-S are genetically associated with a higher risk of developing PCOS. Despite a lack of information on its effects, EPIA-S has recently been proposed as a marker for oral or intramuscular testosterone administration (32, 33). This was also proposed for the administration of other steroid hormones, such as dehydroepiandrosterone (DHEA), 4-androstenedione, and dihydrotestosterone (34). PCOS has long been recognized as a disorder of excessive androgen biosynthesis, use, or metabolism. DHEA sulfate (DHEA-S), DHEA, androstenedione, and testosterone are routinely assessed to identify HA in women with PCOS (35). However, these biochemical indices usually cannot provide a reliable reflection of HA. EPIA-S might be an alternative biomarker in the diagnosis of PCOS, considering its excellent performance in testosterone management. However, the relationship between EPIA-S and PCOS has not been verified by clinical data, and further research is required to understand the potential role of EPIA-S in the diagnosis or treatment of PCOS.

The present study also identified additional metabolites that showed possible association with PCOS, including 3-dehydrocarnitine, 4-hydroxyhippurate, hexadecanedioate, and β-hydroxyisovalerate. 3-Dehydrocarnitine is a member of the carnitine family that is an intermediate in carnitine degradation. Carnitines have long been associated with weight loss, glucose tolerance, insulin function, and fatty acid metabolism (36). A recent study also suggested that 3-dehydrocarnitine is an early biomarker for predicting type 2 diabetes, with applications even prior to the development of insulin resistance (37). Thus, 3-dehydrocarnitine might play a role in abnormal glucose metabolism, which is a common clinical manifestation in PCOS patients. 4-Hydroxyhippuric acid is a microbial end-product derived from polyphenol metabolism by the microflora in the intestine (38). A natural polyphenol, resveratrol, is reported to play a role in inhibiting, androgen production and has been suggested to be a potential therapeutic agent for PCOS (39–41). This might suggest that the polyphenols have a potential value as therapeutic compounds for PCOS. Hexadecanedioate is a candidate biomarker for monitoring organic anion-transporting polypeptide (OATP) function in preclinical species or humans (42). OATP is a group of transporters that are required in DHEA circulation. A previous study also found increased levels of OATP-family transporters in patients with PCOS-endometria, which suggests that OATP plays a functional role in the pathogenesis of PCOS (43). β-Hydroxyisovalerate is a conjugate base of 3-hydroxyisovaleric acid. 3-Hydroxyisovaleric acid was demonstrated to be related to impaired cellular respiration and mitochondrial function (44). Carnitines were also suggested to be involved in the metabolism of 3-hydroxyisovaleric acid (45). In general, the identified metabolites contributed to our understanding of the pathogenesis of PCOS and might also serve as possible therapeutic targets.

We focused on genetic variants that contribute to variation in the target metabolites. The SNP rs13222543, which is located at the intron region of ZCWPW1, was the most significant variant for EPIA-S. ZCWPW1 is a candidate gene for Alzheimer's disease; however, no link had previously been established between ZCWPW1 and EPIA-S (46, 47). Interestingly, enrichment in the male testis for RNA expression of ZCWPW1 has been reported by the Human Protein Atlas database (https://www.proteinatlas.org/), although the expression levels are also high in the thyroid gland, fallopian tube, and ovary (48). This suggests that ZCWPW1 may play a role in steroid hormone metabolism. The SNP rs6691848 is related to 3-dehydrocarnitine and is located on ZMYM6, which is actively expressed in the pituitary gland, and regulates steroid metabolism. Although further evidence is lacking, these connections provide new clues to understand the underlying molecular mechanisms of PCOS.

The present study has several limitations. First, the MR identified causal metabolites associated with the risk of developing PCOS using genetic variants as instrumental variables. Further experimental studies should be conducted to verify these findings. Second, multiple testing was not adjusted, but robustness of the results had been supported by using multiple MR algorithms. Third, the accuracy of MR depends on how well the genetic instruments explain the exposure. The current GWAS analysis on metabolites is based on European populations with a limited sample size. Effort should therefore be made to collect more samples across a broader swath of the population to provide a more accurate assessment of the influence of genetic factors on metabolites. Finally, the findings of our study might be only limited to the European population, not necessarily generalizable to others.

## Conclusions

The present study adopted an MR approach to identify PCOS-related metabolites. The MR approach used genetic variants as instrumental variables to provide unconfounded estimates of the causal relationships between serum metabolites and PCOS. EPIA-S was identified as a causal metabolite that was robustly associated with PCOS development. Some other metabolites, such as 3-dehydrocarnitine, 4-hydroxyhippurate, hexadecanedioate, and β-hydroxyisovalerate, may also have causal effects on the development of PCOS. We emphasized the role of genetic factors underlying the causal relationships between metabolites and PCOS. We provided novel insights by integrating metabolomics with genomics to better understand the mechanisms underlying the pathogenesis of human disease.

## Data Availability Statement

Publicly available datasets were analyzed in this study. This data can be found here: http://metabolomics.helmholtz-muenchen.de/gwas/, https://doi.org/10.17863/CAM.27720.

## Author Contributions

XL and SS were responsible for the study conception and study design. MJ, CH, and QZ were involved in data acquisition and study execution. SS and MJ analyzed the data and drafted the manuscript. WS and JS contributed to interpretation and editing of the manuscript. All authors approved the final version of the manuscript.

## Conflict of Interest

The authors declare that the research was conducted in the absence of any commercial or financial relationships that could be construed as a potential conflict of interest.
